# Future impact of nanotechnology on medicine and dentistry

**DOI:** 10.4103/0972-124X.44088

**Published:** 2008

**Authors:** Mallanagouda Patil, Dhoom Singh Mehta, Sowjanya Guvva

**Affiliations:** 1 *Professor, Department of Periodontics and Implantology, Bapuji Dental College and Hospital, Davanagere, Karnataka, India*; 2 *Professor and Head, Department of Periodontics and Implantology, Bapuji Dental College and Hospital, Davanagere, Karnataka, India*; 3 *Postgraduate Student, Department of Periodontics and Implantology, Bapuji Dental College and Hospital, Davanagere, Karnataka, India*

**Keywords:** Nanodentistry, nanomedicine, nanoscience, nanotechnology

## Abstract

The human characteristics of curiosity, wonder, and ingenuity are as old as mankind. People around the world have been harnessing their curiosity into inquiry and the process of scientific methodology. Recent years have witnessed an unprecedented growth in research in the area of nanoscience. There is increasing optimism that nanotechnology applied to medicine and dentistry will bring significant advances in the diagnosis, treatment, and prevention of disease. Growing interest in the future medical applications of nanotechnology is leading to the emergence of a new field called nanomedicine. Nanomedicine needs to overcome the challenges for its application, to improve the understanding of pathophysiologic basis of disease, bring more sophisticated diagnostic opportunities, and yield more effective therapies and preventive properties. When doctors gain access to medical robots, they will be able to quickly cure most known diseases that hobble and kill people today, to rapidly repair most physical injuries our bodies can suffer, and to vastly extend the human health span. Molecular technology is destined to become the core technology underlying all of 21^st^ century medicine and dentistry. In this article, we have made an attempt to have an early glimpse on future impact of nanotechnology in medicine and dentistry.

## INTRODUCTION

The world began without man, and it will complete itself without him. …Cloude Levi Strauss. Winfred Phillips, DSc, said, “You have to be able to fabricate things, you have to be able to analyze things, you have to be able to handle things smaller than ever imagined in ways not done before”.[1] Many researchers believed that in future, scientific devices that are dwarfed by dust mites may one day be capable of grand biomedical miracles.

The vision of nanotechnology introduced in 1959 by late Nobel Physicist Richard P Faynman in dinner talk said, “There is plenty of room at the bottom,”[2] proposed employing machine tools to make smaller machine tools, these are to be used in turn to make still smaller machine tools, and so on all the way down to the atomic level, noting that this is “a development which I think cannot be avoided”. He suggested nanomachines, nanorobots, and nanodevices ultimately could be used to develop a wide range of automically precise microscopic instrumentation and manufacturing tools, could be applied to produce a vast quantities of ultrasmall computers and various nanoscale microscale robots.

Feynman's idea remained largely undiscussed until the mid-1980s, when the MIT educated engineer K Eric Drexler published “*Engines of Creation*”, a book to popularize the potential of molecular nanotechnology.[3]

Nano comes from the Greek word for dwarf, usually nanotechnology is defined as the research and development of materials, devices, and systems exhibiting physical, chemical, and biological properties that are different from those found on a larger scale (matter smaller than scale of things like molecules and viruses).[4]

Old rules don't apply, small things behave differently. Researchers in nanoland are also making really, really small things with astonishing properties like the carbon nanotube. Chris Papadopoulos, a nanotechnology researcher says, “The carbon nanotube is the poster boy for nanotechnology”. It's is a very thin sheet of graphite that's formed into a tube, its strength can be harnessed by embedding them in constructive materials, among other applications, nanotubes may be part of future improvements for high-performance air craft.

In nanoland, tiny differences in size can add up to huge differences in function. Ted Sergent, author of *The dance of Molecules*, says matter is tunable at nanoscale. For example, change the length of a guitar string and you change the sound it makes; change the size of semiconductors called quantum dots, and you change their rainbow of colors from a single material. Sergent made a three-nanometric dot that ‘glows’ blue, and four nanometer dot that glows red and a five nanometer dot that emits infrared rays or heat.

Nanotechnology will affect everything, says William Atkinson, author of Nanoscom. Nanotechnology and the big changes coming from the inconceivably small. It'll be like a blizzard; snowflakes whose weight you can't detect can bring a city to a standstill. Nanotechnology is going to be like that.

The unique quantum phenomena that happen at the nanoscale, draw researchers from many different disciplines to the field, including medicine, chemistry, physics, engineering, and others (dentistry).

The scientists in the field of regenerative medicine and tissue engineering are continually looking for new ways to apply the principles of cell transplantation, material science, and bioengineering to construct biological substitutes that will restore and maintain normal function in diseased and injured tissue. Development of more refined means of delivering medications at therapeutic levels to specific sites is an important clinical issue, for applications of such technology in medicine, and dentistry.[5]

### Nanomedicine

**Figure 1:**
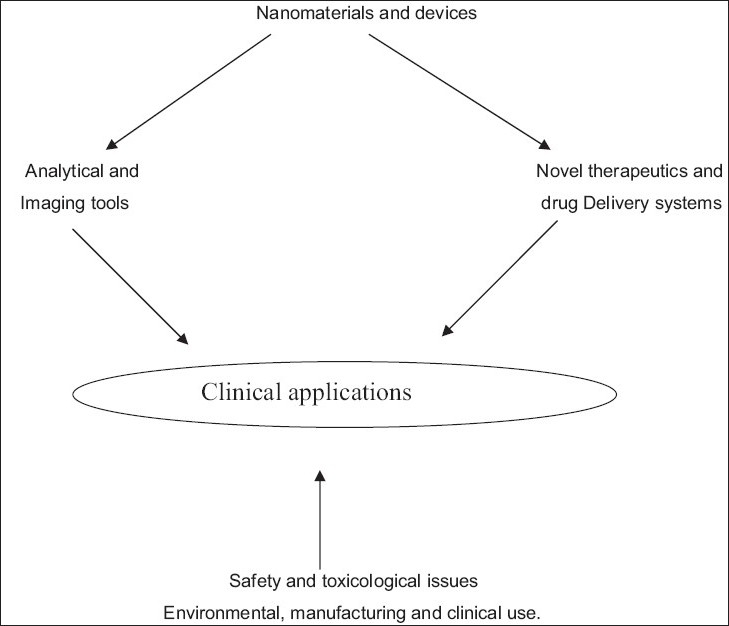
Dimensions in Nanomedicine

The field of “Nanomedicine” is the science and technology of diagnosing, treating, and preventing disease and traumatic injury, of relieving pain, and of preserving and improving human health, using nanoscale structured materials, biotechnology, and genetic engineering, and eventually complex machine systems and nonorobots.[5] It was perceived as embracing five main subdisciplines that in many ways are overlapping by common technical issues [Figure 1].

### Nanodiagnostics

It is the use of nanodevices for the early disease identification or predisposition at cellular and molecular level. In *in-vitro* diagnostics, nanomedicine could increase the efficiency and reliability of the diagnostics using human fluids or tissues samples by using selective nanodevices, to make multiple analyses at subcellular scale, etc. In *in vivo* diagnostics, nanomedicine could develop devices able to work inside the human body in order to identify the early presence of a disease, to identify and quantify toxic molecules, tumor cells.

### Regenerative medicine

It is an emerging multidisciplinary field to look for the reparation, improvement, and maintenance of cells, tissues, and organs by applying cell therapy and tissue engineering methods. With the help of nanotechnology it is possible to interact with cell components, to manipulate the cell proliferation and differentiation, and the production and organization of extracellular matrices.

Present day nanomedicine exploits carefully structured nanoparticles such as dendrimers, carbon fullerenes (buckyballs), and nanoshells to target specific tissues and organs. These nanoparticles may serve as diagnostic and therapeutic antiviral, antitumor, or anticancer agents. Years ahead, complex nanodevices and even nanorobots will be fabricated, first of biological materials but later using more durable materials such as diamond to achieve the most powerful results.[6]

The human body is comprised of molecules, hence the availablity of molecular nanotechnology will permit dramatic progress to address medical problems and will use molecular knowledge to maintain and improve human health at the molecular scale.

### Applications in medicine

Within 10–20 years it should become possible to construct machines on the micrometer scale made up of parts on the nanometer scale. Subassemblies of such devices may include such as useful robotic components as 100 nm manipulater arms, 10 nm sorting rotors for molecule by molecule reagent purification, and smooth super hard surfaces made of automically flawless diamond.

Nanocomputers would assume the important task of activating, controlling, and deactivating such nanomechanical devices. Nanocomputers would store and execute mission plans, receive and process external signals and stimuli, communicate with other nanocomputers or external control and monitoring devices, and possess contextual knowledge to ensure safe functioning of the nanomechanical devices. Such technology has enormous medical and dental implications.

Programmable nanorobotic devices would allow physicians to perform precise interventions at the cellular and molecular level. Medical nanorobots have been proposed for genotological[7] applicatons in pharmaceuticals research,[8] clinical diagnosis, and in dentistry,[9] and also mechanically reversing atherosclerosis, improving respiratory capacity, enabling near-instantaneous homeostasis, supplementing immune system, rewriting or replacing DNA sequences in cells, repairing brain damage, and resolving gross cellular insults whether caused by irreversible process or by cryogenic storage of biological tissues.

Feynman offered the first known proposal for a nanorobotic surgical procedure to cure heart disease,[2] “A friend of mine (Albert R. Hibbs) suggests a very interesting possibility for relatively small machines. He says that, although it is a very wild idea, it would be interesting in surgery if you could swallow the surgeon. You put the mechanical surgeon inside the blood vessel and it goes into the heart and looks around. It finds out which valve is the faulty and takes a little knife and slices it out, that we can manufacture an object that maneuvers at that level, other small machines might be permanently incorporated in the body to assist some inadequately functioning organs”.[2]

Many disease causing culprits such as bacteria and viruses are nanosize. So, it only makes sense that nanotechnology would offer us ways of fighting back. The ancient greeks used silver to promote healing and prevent infection, but the treatment took backseat when antibiotics came on the scene. Nycryst pharmaceuticals (Canada) revived and improved an old cure by coating a burn and wound bandage with nanosize silver particles that are more reactive than the bulk form of metal. They penetrate into skin and work steadily. As a result, burn victims can have their dressings changed just once a week.

Genomics and protomics research is already rapidly elucidating the molecular basis of many diseases. This has brought new opportunities to develop powerful diagnostic tools able to identify genetic predisposition to diseases. In the future, point of care diagnosis will be routinely used to identify those patients requiring preventive medication to select the most appropriate medication for individual patients, and to monitor response to treatment. Nanotechnology has a vital role to play in realizing cost-effective diagnostic tools.

Chris Backous developing Lab–on-Chip to give doctor immediate results from medical tests for cancer and viruses, it gets its information by analyzing the genetic material in individual cells. Advances in gene sequencing mean this can now be done quickly and sequencing with tiny samples of body fluids or tissues such as blood, bone marrow, or tumors. The device can also detect the *BK* virus, a sign of trouble in patients who have had kidney transplants. Ultimately (Pilarski thinks,) chip technology will be able to detect what kind of flu a person has, or, even if they have SARS or HIV.

Nanotechnology has the potential to offer invaluable advances such as use of nanocoatings to slow the release of asthma medication in the lungs, allowing people with asthma to experience longer periods of relief from symptoms after using inhalants. Thus, what nanotechnology tries to do is essentially make drug particles in such a way, that they don't dissolve that fast, done this with.

Nanosensors developed for military use in recognizing airborne rogue agents and chemical weapons to detect drugs and other substances in exhaled breath.[1] Basically, you can detect many drugs in breath, but the amount you detect in breath is going to be related to the amount that you take and also to whether it partitions well between the blood and the breath. Drug abuse like marijuna (and things like), concentration of alcohol, testing of athletes for banned substances, and individual's drug treatment programs are two areas long overdue for breath detection technologies. We see this in future totally replacing urine testing.

Currently, most legal and illegal drug overdoses have no specific way to be effectively neutralized, using nanoparticles as absorbents of toxic drugs, is another area of medical nanoscience that is rapidly gaining momentum. Goal is design nanostructures that effectively bind molecular entities, which currently don't have effective treatments. We are putting nanosponges into the blood stream and they are soaking up toxic drug molecules to reduce the free amount in the blood, in turn, causes a resolution of the toxicity that was there before you put the nanosponges into the blood.

French and Italian researchers have come up with a completely new approach to render anticancer and antiviral nucleoside analoges significantly more potent. By linking the nucleoside analoges to sequalene, a biochemical precursor to the whole family of steroids, the researchers observed the self-organization of amphiphilic molecules in water. These nanoassemblies exhibited superior anticancer activity *in vitro* in human cancer cells.

Laurie B Gower, PhD, has been researching bone formation and structure at the nanoscale level. She is examining biomimetic methods of constructing a synthetic bone graft substitute with a nanostructured architecture that matches natural bone so that it would be accepted by the body and guide the cells toward the mending of damaged bones. Biomineralization refers to minerals that are formed biologically, which have very different properties than geological minerals or lab-formed crystals. The crystal properties found in bone are manipulated at nanoscale and are imbedded within collagen fibers to create an interpenetrating organic–inorganic composite with unique mechanical properties. She foresees numerous implications of the material in the future of osteology.

Hichan Fenniri, a chemistry professor, tried to make artificial joints act more like natural ones. Fenniri has made a nanotube coating for titanium hip or knee, is very good mimic of collagen, as a result of coating attracts and attaches more bone cells, osteoblasts, which help in bone growth quickly than uncoated hip or knee.

There is ongoing attempts to build ‘medical microrobots’ for *in vivo* medical use.[10] In 2002, Ishiyama *et al*,[11] at Tohku University developed tiny magnetically driven spinning screws intended to swim along veins and carry drugs to infected tissues or even to burrow into tumors and kill them with heat. In 2005, Brad Nelson's[12] team reported the fabrication of a microscopic robot, small enough (approximately 200 µm) to be injected into the body through a syringe. They hope that this device or its descendants might someday be used to deliver drugs or perform minimally invasive eye surgery. Gorden's[9,13] group at the University of Manitoba has also proposed magnetically controlled ‘cytobots’ and ‘karyobots’ for performing wireless intracellular and intranuclear surgery.

‘Respirocytes’, the first theoreotical design study of a complete medical nanorobot ever published in peer-reviewed journal described a hypothetical artificial mechanical red blood cell or ‘respirocyte’ made of 18 billion precisely arranged structural atoms.[10,14] The respirocyte is a bloodborne spherical 1 µm diamondedoid 1000 atmosphere pressure vessel with reversible molecule selective surface pumps powered by endogenous serum glucose. This nanorobot would deliver 236 times more oxygen to body tissues per unit volume than natural red cells and would manage carbonic acidity, controlled by gas concentration sensors and an onboard nanocomputer.

### Nanorobotic microbivores

Artificial phagocytes called microbivores could patrol the bloodstream, seeking out and digesting unwanted pathogens including bacteria, viruses, or fungi.[10,15] Microbivores would achieve complete clearance of even the most severe septicemic infections in hours or less. The nanorobots do not increase the risk of sepsis or septic shock because the pathogens are completely digested into harmless sugars, amino acids, and the like, which are the only effluents from the nanorobot.

### Surgical nanorobotics

A surgical nanorobot, programmed or guided by a human surgeon, could act as a semiautonomous on site surgeon inside the human body, when introduced into the body through vascular system or cavities. Such a device could perform various functions such as searching for pathology and then diagnosing and correcting lesions by nanomanipulation, coordinated by an onboard computer while maintaining contact with the supervising surgeon via coded ultrasound signals.[10]

The earliest forms of cellular nanosurgery are already being explored today. For example, rapidly vibrating (100 Hz) micropipette with a <1 µm tip diameter has been used to completely cut dentrites from single neurons without damaging cell viability.[16] Axotomy of roundworm neurons was performed by femtosecond laser surgery, after which the axons functionally regenerated.[17] Femtolaser acts like a pair of nanoscissors by vaporizing tissue locally while leaving adjacent tissue unharmed. Femtolaser surgery has performed the individual chromosomes.[18]

### Nanogenerators'

They could make new class of self-powered implantable medical devices, sensors, and portable electronics, by converting mechanical energy from body movement, muscle stretching, or water flow into electricity.

Nanogenerators produce electric current by bending and then releasing zinc oxide nanowires, which are both piezoelectric and semiconducting. Nanowires can be grown on polymer-based films, use of flexible polymer substrates could one day allow portable devices to be powered by movement of their users.

“Our bodies are good at converting chemical energy from glucose into the mechanical energy of our muscles,” Wang (faculty at Peking University and National Center for Nanoscience and Technology of China) explained “these nanogenerators can take mechanical energy and convert it to electrical energy for powering devices inside the body. This could open up tremendous possibilities for self-powered implantable medical devices.”

### Nanodentistry

Nanodentistry will make possible the maintenance of comprehensive oral health by employing nanomaterials, biotechnology, including tissue engineering, and ultimately, dental nanorobotics. New potential treatment opportunities in dentistry may include, local anesthesia, dentition renaturalization, permanent hypersensitivity cure, complete orthodontic realignments during a single office visit, covalently bonded diamondised enamel, and continuous oral health maintenance using mechanical dentifrobots.

When the first micro-size dental nanorobots can be constructed, dental nanorobots might use specific motility mechanisms to crawl or swim through human tissue with navigational precision, acquire energy, sense, and manipulate their surroundings, achieve safe cytopenetration and use any of the multitude techniques to monitor, interrupt, or alter nerve impulse traffic in individual nerve cells in real time.

These nanorobot functions may be controlled by an onboard nanocomputer that executes preprogrammed instructions in response to local sensor stimuli. Alternatively, the dentist may issue strategic instructions by transmitting orders directly to *in vivo* nanorobots via acoustic signals or other means.

### Inducing anesthesia

One of the most common procedure in dental practice, to make oral anesthesia, dental professionals will instill a colloidal suspension containing millions of active analgesic micron-sized dental nanorobot ‘particles’ on the patient's gingivae. After contacting the surface of the crown or mucosa, the ambulating nanorobots reach the dentin by migrating into the gingival sulcus and passing painlessly through the lamina propria or the 1–3-micron thick layer of loose tissue at the cementodentinal junction. On reaching dentin, the nanorobots enter dentinal tubules holes that are 1–4 microns in diameter and proceed toward the pulp, guided by a combination of chemical gradients, temperature differentials, and even positional navigation, all under the control of the onboard nanocomputer as directed by the dentist.[9]

There are many pathways to choose from, near to CEJ, midway between junction and pulp, and near to pulp. Tubules diameter increases as it nears the pulp, which may facilitate nanorobot movement, although circumpulpal tubule openings vary in numbers and size (tubules number density 22,000 mm DEJ, 37,000 mm square midway, ans 48000 mm square near to pulp). Tubules branching patterns, between primary and irregular secondary dentin, regular secondary dentin in young and old teeth (sclerosing) may present a significant challenge to navigation.

The presence of natural cells that are constantly in motion around and inside the teeth including human gingival and pulpal fibroblasts, cementoblasts of the CDJ, bacteria inside dentinal tubules, odontoblasts near the pulp dentin border, and lymphocytes within the pulp or lamina propria suggested that such journey should be feasible by cell-sized nanorobots of similar mobility.

Once installed in the pulp and having established control over nerve impulse traffic, the analgesic dental nanorobots may be commanded by the dentist to shut down all sensitivity in any particular tooth that requires treatment. When on the hand-held controller display, the selected tooth immediately becomes numb. After the oral procedures completed, the dentist orders the nanorobots to restore all sensation, to relinguish control of nerve traffic and to engress, followed by aspiration. Nanorobotic analgesics offer greater patient comfort and reduced anxiety, no needles, greater selectivity, and controllability of the analgesic effect, fast and completely reversible switchable action and avoidance of most side effects and complications.

### Tooth repair

Nanorobotic manufacture and installation of a biologically autologous whole replacement tooth that includes both mineral and cellular components, that is, ‘complete dentition replacement therapy’ should become feasible within the time and economic constraints of a typical office visit through the use of an affordable desktop manufacturing facility, which would fabricate the new tooth in the dentist's office.

Chen *et al*[19] took advantage of these latest developments in the area of nanotechnology to simulate the natural biomineralization process to create the hardest tissue in the human body, dental enamel, by using highly organized microarchitectural units of nanorod-like calcium hydroxyapatite crystals arranged roughly parallel to each other.

### Dentin hypersensitivity

Natural hypersensitive teeth have eight times higher surface density of dentinal tubules and diameter with twice as large than nonsensitive teeth. Reconstructive dental nanorobots, using native biological materials, could selectively and precisely occlude specific tubules within minutes, offering patients a quick and permanent cure.[9]

### Tooth repositioning

Orthodontic nanorobots could directly manipulate the periodontal tissues, allowing rapid and painless tooth straightening, rotating and vertical repositioning within minutes to hours.

### Tooth renaturalization

This procedure may become popular, providing perfect treatment methods for esthetic dentistry. This trend may begin with patients who desire to have their (1) old dental amalgams excavated and their teeth remanufactured with native biological materials, and (2) full coronal renaturalization procedures in which all fillings, crowns, and other 20^th^ century modifications to the visible dentition are removed with the affected teeth remanufactured to become indistinguishable from original teeth.

### Dental durability and cosmetics

Durability and appearance of tooth may be improved by replacing upper enamel layers with covalently bonded artificial materials such as sapphire or diamond,[20] which have 20–100 times the hardness and failure strength of natural enamel or contemporary ceramic veneers and good biocompatibility. Pure sapphire and diamond are brittle and prone to fracture, can be made more fracture resistant as part of a nanostructured composite material that possibly includes embedded carbon nanotubes.

Nanorobotic dentifrice (dentifrobots) delivered by mouthwash or toothpaste could patrol all supragingival and subgingival surfaces at least once a day metabolizing trapped organic mater into harmless and odorless vapors and performing continous calculus debridement.

Properly configured dentifrobots could identify and destroy pathogenic bacteria residing in the plaque and elsewhere, while allowing the 500 species of harmless oral microflora to flourish in a healthy ecosystem. Dentifrobots also would provide a continous barriers to halitosis, since bacterial putrification is the central metabolic process involved in oral malodor. With this kind of daily dental care available from an early age, conventional tooth decay and gingival deseases will disappear into the annals of medical history.

Potential benefits of nanotechnology are its ability to exploit the atomic or molecular properties of materials and the development of newer materials with better properties. Nanoproducts can be made by: building-up particles by combining atomic elements and using equipments to create mechanical nanoscale objects.

Nanotechnology has improved the properties of various kinds of fibers.[21] Polymer nanofibers with diameters in the nanometer range, possess a larger surface area per unit mass and permit an easier addition of surface functionalities compared to polymer microfibers.[21,22] Polymer nanofiber materials have been studied as drug delivery systems, scaffolds for tissue engineering and filters. Carbon fibers with nanometer diamensions showed a selective increase in osteoblast adhesion necessary for successful orthopedic/dental implant applications due to a high degree of nanometer surface roughness.[23]

Nonagglomerated discrete nanoparticles are homogenously manufactured in resins or coatings to produce nanocomposites. The nanofiller used include an aluminosilicate powder having a mean particles size of about 80 nm and 1:4 M ratio of alumina to silica. Advantages – superior hardness, flexible strength, modulus of elasticity, translucency and esthetic appeal, excellent color density, high polish, and polish retention, and excellent handling properties.[24] (Filtek O supreme Univrasl Restorative Pure Nano O).

Heliometer, microfilled composite resin, a close examination of this composite suggests that a form of nanotechnology was in use years ago, yet never recognized.

Nanosolutions produce unique and dispersible nanoparticles that can be added to various solvents, paints, and polymers in which they are dispersed homogenously. Nanotechnology in bonding agents ensures homogeneity and so the operator can now be totally confident that the adhesive is perfectly mixed every time.

Nanofillers are integrated in the vinylsiloxanes, producing a unique addition siloxane impression material. Better flow, improved hydrophilic properties, hence fewer voids at margin and better model pouring, enhanced detail precision.[25]

## DISCUSSION

Nanotechnology is part of a predicted future in which dentistry and periodontal practice may become more high-tech and more effective looking to manage individual dental health on a microscopic level by enabling us to battle decay where it begins with bacteria. Construction of a comprehensive research facility is crucial to meet the rigorous requirements for the development of nanotechnologies.

Researchers are looking at ways to use microscopic entities to perform tasks that are now done by hand or with equipment. This concept is known as nanotechnology. Tiny machines, known as nanoassemblers, could be controlled by computer to perform specialized jobs. The nanoassemblers could be smaller than a cell nucleus so that they could fit into places that are hard to reach by hand or with other technology. Used to destroy bacteria in the mouth that cause dental caries or even repair spots on the teeth where decay has set in, by use of computer to direct these tiny workers in their tasks.

Nanotechnology has tremendous potential, but social issues of public acceptance, ethics, regulation, and human safety must be addressed before molecular nanotechnology can be seen as the possibility of providing high quality dental care to the 80% of the world's population that currently receives no significant dental care.

Role of periodontitis will continue to evolve along the lines of currently visible trends. For example, simple self-care neglect will become fewer, while cases involving cosmetic procedures, acute trauma, or rare disease conditions will become relatively more commonplace.

Trends in oral health and disease also may change the focus on specific diagnostic and treatment modalities. Increasingly preventive approaches will reduce the need for cure prevention a viable approach for the most of them.

Diagnosis and treatment will be customized to match the preferences and genetics of each patient. Treatment options will become more numerous and exciting. All this will demand, even more so than today, the best technical abilities, professional skills that are the hallmark of the contemporary dentist and periodontist. Developments are expected to accelerate significantly.

Nanometers and nanotubes, technologies could be used to administer drugs more precisely. Technology should be able to target specific cells in a patient suffering from cancer or other life-threatening conditions. Toxic drugs used to fight these illnessess would become much more direct and consequently less harmful to the body.

## CONCLUSION

The visions described in this article may sound unlikely, implausible, or even heretic. Yet, the theoretical and applied research to turn them into reality is progressing rapidly. Nanotechnology will change dentistry, healthcare, and human life more profoundly than many developments of the past. As with all technologies, nanotechnology carries a significant potential for misuse and abuse on a scale and scope never seen before. However, they also have potential to bring about significant benefits, such as improved health, better use of natural resources, and reduced environmental pollution. These truly are the days of miracle and wonder.

Current work is focused on the recent developments, particularly of nanoparticles and nanotubes for periodontal management, the materials developed from such as the hollow nanospheres, core shell structures, nanocomposites, nanoporous materials, and nanomembranes will play a growing role in materials development for the dental industry.

Once nanomechanics are available, the ultimate dream of every healer, medicine man and physician throughout recorded history will, at last become a reality. Programmable and controllable microscale robots comprised of nanoscale parts fabricated to nanometer precision will allow medical doctors to execute curative and reconstructive procedures in the human body at the cellular and molecular levels. Nanomedical physicians of the 21^st^ century will still make good use of the body's natural healing powers and homeostatic mechanisms, because all else equal, those interventions are best that intervene least.
